# A Cause of Disease

**Published:** 1890-06

**Authors:** 


### A CAUSE OF DISEASE.

Constipation is very generally considered quite an unimportant trouble, and at the most merely capable of causing but a few uncomfortable symptoms, such as headache, malaise, etc. This popular idea is an erroneous one, for to constipation can be attributed a wide range of really very serious affections. It acts in this way. In the matters retained in the intestines there are poisons, and some of them very virulent. They are absorbed in the intestines and distributed over the body. Their most marked effect, as far as we know, is upon the nervous system, which they may be said to intoxicate, or, in other words, excite to a great degree. One of the effects of these poisons is insomnia, and really they very frequently are the sole cause of that distressing affection. Then there is chlorosis, or "green sickness," a disorder of girls, usually between 14 and 18 years of age. That, undoubtedly, is frequently caused indirectly by constipation and reabsorption of the poisons in question. Again, insanity may be included among the consequences of constipation. Dr. Moyer gives the history of three typical cases of insanity which occurred in his practice alone, and which were entirely relieved by large injections. Similar cases have been reported by others. One eminent physician suffering from constipation had ample opportuity to study some of its pernicious effects. On one occasion hallucination and phantasms appeared to him and continued for three days. He took a large injection and so removed the cause, and immediately his mind cleared up. He testified that there is no distinguishing symptom of this form of poisoning, but an intellectual disturbance, characterized by a peculiar depression of spirit, by anguish of mind, and by self-accusations of wickedness and baseness. Probably physicians as yet do not know all the evil effects of constipation, and, doubtless a large number of affections remain to be added to the list of consequences.

